# The Influence of pH Values on the Rheological, Textural and Release Properties of Carbomer Polacril^®^ 40P-Based Dental Gel Formulation with Plant-Derived and Synthetic Active Components

**DOI:** 10.3390/molecules25215018

**Published:** 2020-10-29

**Authors:** Yuliia Maslii, Olena Ruban, Giedre Kasparaviciene, Zenona Kalveniene, Anna Materiienko, Liudas Ivanauskas, Agne Mazurkeviciute, Dalia M. Kopustinskiene, Jurga Bernatoniene

**Affiliations:** 1 Department of Industrial Technology of Drugs, National University of Pharmacy, 61002 Kharkiv, Ukraine; julia.masliy@gmail.com (Y.M.); ruban_elen@ukr.net (O.R.); 2 Department of Drug Technology and Social Pharmacy, Lithuanian University of Health Sciences, LT-50161 Kaunas, Lithuania; Giedre.Kasparaviciene@lsmuni.lt (G.K.); Zenona.Kalveniene@lsmuni.lt (Z.K.); 3 Department of Quality, Standardization and Certification of Drugs, National University of Pharmacy, 61002 Kharkiv, Ukraine; anna.materienko@gmail.com; 4 Department of Analytical Chemistry, Lithuanian University of Health Sciences, LT-50161 Kaunas, Lithuania; Liudas.Ivanauskas@lsmuni.lt; 5 Department of Clinical Pharmacy, Lithuanian University of Health Sciences, LT-50161 Kaunas, Lithuania; Agne.Mazurkeviciute@lsmuni.lt; 6 Institute of Pharmaceutical Technologies, Medical Academy, Lithuanian University of Health Sciences, LT-50161 Kaunas, Lithuania; DaliaMarija.Kopustinskiene@lsmuni.lt

**Keywords:** periodontal tissue, oral mucosa diseases, Carbomer Polacril^®^ 40P-based gel, pH, rheological studies, textural analysis, release test

## Abstract

The physicochemical properties, especially pH value of dental medicines, have significant influence on the health of oral cavity tissues. The pH of formulations should correspond to the value of saliva pH (5.5–8.0). For carbomer-based gels, the required pH value is obtained by neutralizing them with alkaline components, which leads to their structuring (thickening). This affects the physical properties of the gel, its residence time at the application site and the rate of release of active pharmaceutical ingredient. Therefore, the main purpose of this study is to evaluate the rheological, textural, and biopharmaceutical properties of Carbomer Polacril^®^ 40P-based dental gel depending on the pH value. Evaluation of the rheological properties of gel preparations were performed by measuring the structural viscosity of the samples as a function of pH and temperature. The textural properties of the gel were evaluated by performing tests regarding back extrusion and spreadability. Carbomer Polacril^®^ 40P-based gels haven’t shown noticeable thixotropic behavior, and were characterized by plastic flow in the whole studied pH range. The structural viscosity at the selected average pH value hasn’t differed at storage (25 °C) and application (37 °C) temperature. Texture studies of dental gels have shown a strong correlation with rheoparameters. Their rheological behavior and textural properties haven’t changed significantly between the pH range of 5.5–6.6. The relatively narrow range of working pH values does not affect the change in the viscosity of the preparation significantly and, consequently, does not affect the release of APIs from the developed Carbomer Polacril^®^ 40P-based dental gel.

## 1. Introduction

Oral cavity diseases are among the most common worldwide. They remain not only a medical but also a social problem [1,2,3,4]. To date, there is a high incidence of periodontal tissue disease among patients of different age groups, which deteriorates the health of other organs within the body, therefore decreasing the social and psychological state of human life [5,6,7,8]. All this, in turn, requires appropriate treatment, significantly addressed through the application of locally acting drugs [9,10].

Gels are the most progressive dosage form in dental practice, which has a high therapeutic efficacy and good consumer properties due to: (a) acceptable viscosity, which prevents rapid washout by saliva, improving the diffusion of active pharmaceutical ingredients (APIs), which enables the individual to maintain an optimal concentration in the application area; (b) high adhesive properties, which allows them to have good distribution on the mucous membrane, providing close contact with tissues and high bioavailability of API; (c) the optimal composition of excipients and the above factors, which provides immediate or prolonged action as needed [11,12,13,14].

We developed a new dental gel formulation, containing a combination of APIs of natural and synthetic origin, for the purpose of treatment and prevention of inflammatory diseases of the periodontium and oral mucosa as well as the adaptation for removable dentures. This formulation provides a multidirectional action on the soft tissues of the oral cavity [15,16]. As a result of previous studies, gelling agent Carbomer Polacril^®^ 40P at a concentration of 1.5%, which is a pharmacopoeial excipient approved for oral administration, was chosen for the development of this gel [17,18]. Carbomers allow to obtain highly-viscous gels of good quality even at low concentrations of a substance-former. They are known for their excellent bioadhesive, thermostable, and organoleptic properties, and therefore, these gelling agents are attractive from both the pharmaceutical and consumer point of view [19,20,21]. In addition, carbomers are compatible with many active ingredients and allow to achieve the required pH value [22,23].

The pH level of dental formulations is of crucial importance to avoiding the disruption of the acid-base balance in the mouth which could negatively impact on the tissues of the oral cavity and the state of the organism as a whole [24,25,26,27]. Therefore, for dental drugs it is necessary to keep this indicator within rational limits, which corresponds to the saliva pH value which, depending on a variety of factors, ranges between 5.5 to 8.0 [28,29,30].

For carbomer-based gels, which are derivatives of acrylic acid, the required pH is obtained through neutralization: with increasing pH value, the globules of carbomer molecules unfold into a bulk network, creating a structure formation which increases the viscosity of the system. These changes affect the ease of use, residence time at the application site, diffusion, the rate of API release, and the physicochemical stability of the composition [22,31]. Therefore, the purpose of our work is to study the rheological, textural and release properties as a function of pH of the Carbomer Polacril^®^ 40P-based dental gel.

## 2. Results and Discussion

There are many studies on the change of rheological properties of carbomer-based gels depending on the pH value, where the use of a variety of carbomers and corresponding neutralizers are considered. However, many of these academic studies focus on gels used for external dermal application [22,32,33,34,35].

Our rheological, texture and biopharmaceutical research is dedicated to the development of a combined mucoadhesive dental gel based on Carbomer Polacril^®^ 40P. In addition to flow and viscosity, an important parameter in the development was the evaluation of the cohesive-adhesive properties of the gel at different pH values in terms of the physiological aspects of its use.

Analysis of existing dental gels established a wide range of pH values: “Metrodent^®^” (Encube Ethicals Private Limited, India)—pH = 4.0–6.5; “Cholisal” (Pharmaceutical Works Jelfa S.A., Poland) —pH = 4.5–6.5; “Metrogyl Denta^®^” (Unique Pharmaceutical Laboratories, India)—pH = 5.0–7.0; “Dentinox-Gel N” (Dentinox Gesellschaft für Pharmaceuticals, Praparate Lenk & Schuppan, Germany)—pH = 6.0–6.5, “Kamistad^®^” (Stada Arzneimittel AG, Germany)—pH = 7.0–8.5. In order to determine a rational pH value for our developed gel, model samples neutralized to different pH in the range of 5.0 to 7.5 were prepared. This interval corresponds to physiological norms for the pH of the oral cavity. Based on existing analysis available in the pharmaceutical market dental gels, a neutralizing agent consisting of solution of sodium hydroxide (10%) was chosen, due to it’s lower toxicity when compared with other adjuvants of this group.

**Figure 1:**
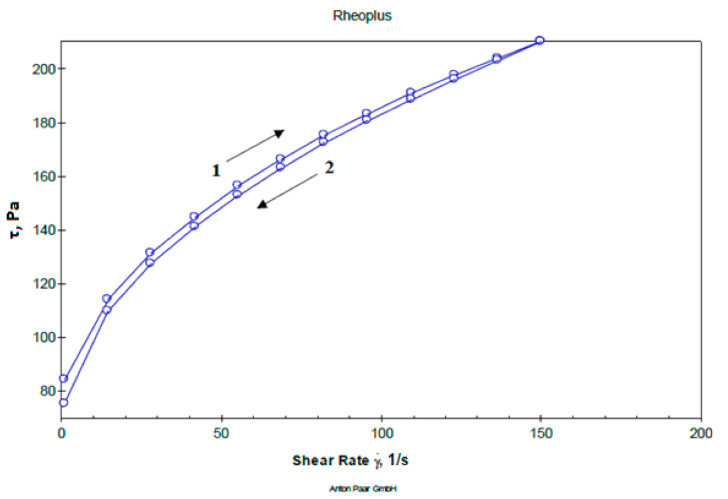
Representative rheogram of dental gel at pH 6.6 (1—up and 2—down curve).

Determination of flow curves and structural viscosity of the studied gel samples were performed within a shear rate range between 0 to 150 s^–1^. The obtained sample of gels, though consisting of differing pH values, all have a plastic type of flow, and are characterized by an insignificant hysteresis loop, i.e., minimal thixotropy, which is a feature of carbomers-based gels and low content of hydrophilic non-aqueous solvents. A representative rheogram of the studied dental gel is presented in Figure 1.

**Figure 2:**
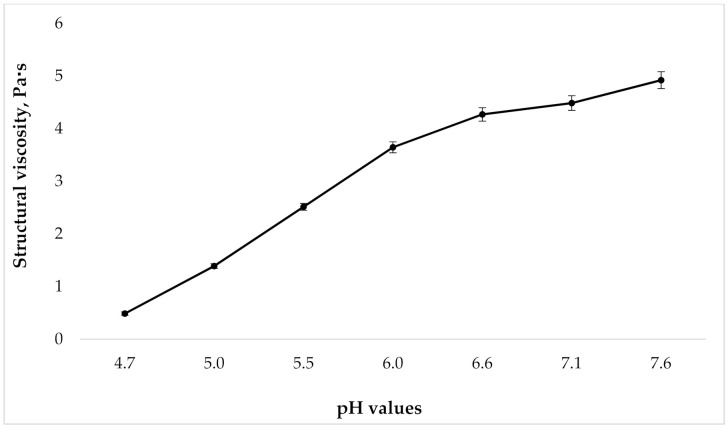
Dependence of structural viscosity on gel pH at shear rate 41.6 s^−1^ (*n* = 3).

Because the gels viscosity changes with varying degrees of carbomers neutralization, the next step in our research was to study the dependence of the structural viscosity of the gel on the pH value. Rheological analysis was performed according to the requirements of European Pharmacopoeia (Ph.Eur.) (current edition) via rotational coaxial cylinder viscometer at 25 °C and a shear rate of D_r_ = 41.6 s^−1^. This value was chosen because it is close to the mixing speed of the gel under industrial conditions. The results are presented in Figure 2.

As can be seen in Figure 2, as the pH grows, the structural viscosity of the gel increases. At the same time, the change in pH from 4.7 to 6.0 leads to a marked increase in viscosity; with a further growth in the pH value, there is no significant rise in the studied indicator. However, given the presence of the tincture “Phytodent”, that contains flavonoids, in the drug composition, from the point of view of ensuring the stability of API, it is important to create a pH that, on the one hand, will not be destructive to them, but on the other hand, will be high enough for prolonged retention of the gel on the surface of the application site. With this in mind, samples with a pH of 5.5 to 6.6 were used for the further studies.

**Figure 3:**
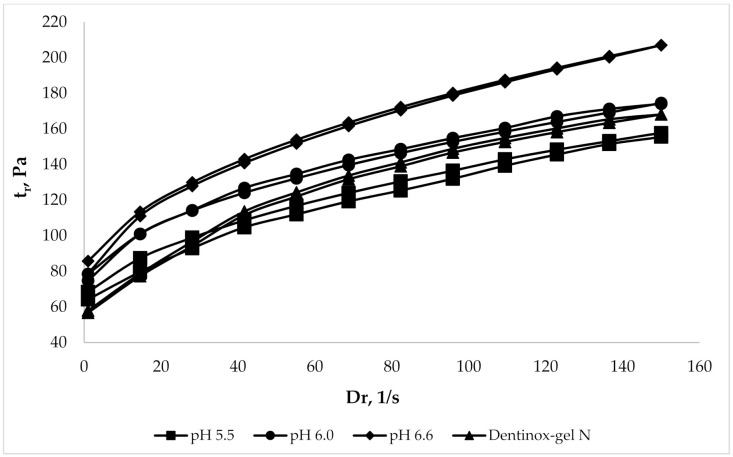
Comparison rheological parameters of gel samples with “Dentinox-gel N”.

To evaluate the rheological parameters of the chosen gel samples in comparison with commercially available products the rheograms were constructed, which represent the dependence of the shear stress (τ) on shear rate (D_r_). The analysis was performed in comparison with the dental preparation “Dentinox-gel N”, which is also made on a carbomer, has a similar composition of API (contains a tincture of medicinal plant raw materials and lidocaine hydrochloride), and has a pH range of 6.0–6.5. The results of analysis, which was made using viscometer with coaxial cylinders at 25 °C, are shown in Figure 3.

Rheograms, which are presented in Figure 3, had shown the rheological similarity of the gel samples with pH range 5.5–6.6 to the compared “Dentinox-gel N” preparation. The sample with maximal pH 6.6 was slightly different from the other gels by more viscous structure.

Flow characteristics at 25 °C (storage temperature) and 37 °C (application temperature) were determined by the Ostwald de Waele rheological model which describes plastic or shear-thickening behavior: *τ = Kγ^n^*, where *τ* is shear stress (Pa), *K* is consistency index (Pa·s^n^), *γ* is shear rate (s^−1^), *n* is flow behavior index (dimensionless). The consistency index is relevant for liquid consistency: if *n* = 1, the fluid is Newtonian and the parameter *K* has the value of Newtonian viscosity *η*. The flow behavior index is a deviation from Newtonian behavior. If *n* < 1, the viscosity decreases with the increasing shear rate, which is typical for plastic systems [36,37,38]. The results are presented in Table 1.

According to the results presented in Table 1, all samples had the flow behavior index from 0.12 to 0.14, which indicates the plastic nature of their flow. The flow behavior index decreases in accordance with increasing gel pH values; the consistency index, on the contrary, increases, which is due to changes in the viscosity of the system. In addition, statistically significant differences were not observed between the rheoparameters with increasing temperature, which proves the thermostability of carbomer-based gel systems.

**Figure 4:**
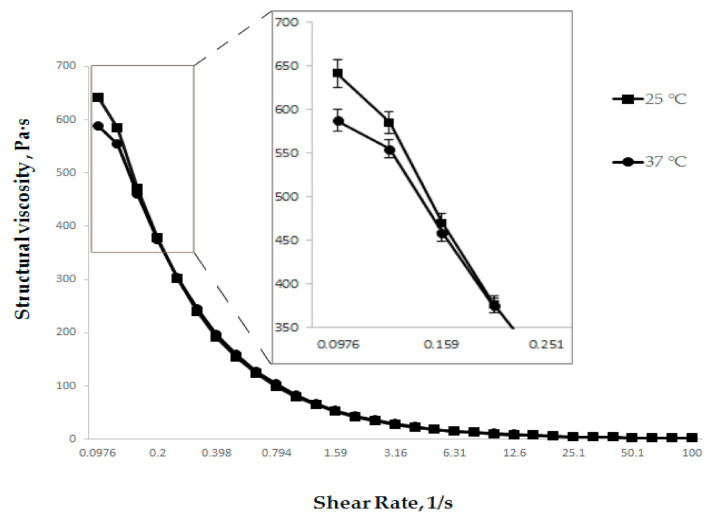
Dependence of structural viscosity on shear rate at different temperatures (*n* = 3).

Additionally, the dependence of the structural viscosity of the developed gel on the temperature at pH 6.0, i.e., in the middle of the selected range, was studied (Figure 4).

As evidenced by the data presented in Figure 4, the viscosity of the developed gel at a storage temperature of 25 °C and a use temperature of 37 °C is almost no different. This indicates the absence of the temperature change effect on the structural viscosity of the gel in the storage/use temperature range.

As the textural properties also affect the consumption characteristics of the gel and, as a result, the effectiveness of the therapy [39,40,41], one of the goals of this research was to study the textural properties of test gel samples, namely cohesiveness, adhesiveness, and firmness. Therefore, it was important to evaluate the effect of each pH value on the texture properties of the developed semi-solid formulation. The studies were performed using a Texture Analyzer.

**Figure 5:**
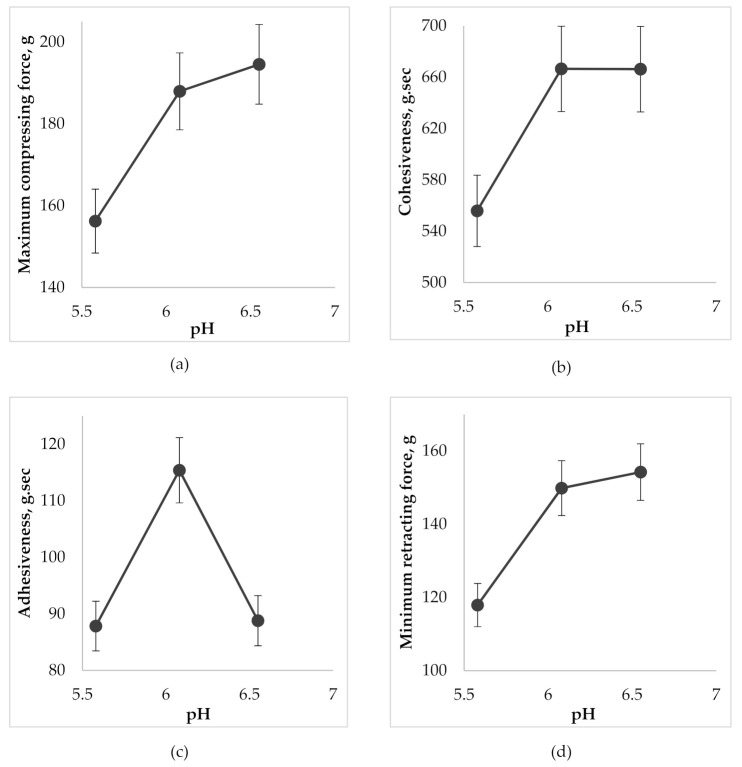
The effect of pH on the gel characteristics during the determination of back extrusion: (**a**) on maximum compressing force; (**b**) on cohesiveness; (**c**) on adhesiveness; (**d**) on minimum retracting force.

Dental gels should have a structure that can withstand the physiological damage caused by jaw movement and saliva flow, while at the same time provide intimate and sustainable contact with oral tissues; that is, it is necessary to maintain a balance between adhesiveness and cohesiveness of the gel [41]. Back extrusion analysis can help to determine these properties. The results of the back extrusion test on the gels at different pH levels are presented in Figure 5.

According to Figure 5a, the increase of gel pH in the selected range from 5.5 to 6.6 caused a growth of compressing force which created a deformation of the gel with further back extrusion. Accordingly, this can be explained by the increase in the firmness of the gel by increasing its viscosity as a result of the formation of a more common 3D-net of thickener molecules and strengthening the bond between them during neutralization (cohesion). Cohesiveness ensures uniform distribution of the gel on the oral mucosa and prevents its rapid washout by saliva, maintaining the gel structure for a longer time. The highest cohesiveness was evident in gel samples with pH 6.0 and 6.6 (Figure 5b). According to the results of Figure 5c the gel with pH 6.0 had the best adhesiveness. The sample with pH 5.5 had a more liquid texture, yet the sample of pH 6.6 is more viscous, which is also proved by rheological studies and, in turn, negatively affected the “surface-gel” contact area and led to a decrease in adhesiveness. When a probe was lifted above the surface of the gel, the sample stretches to form a thread that detaches from the disk at the specified distance. As a result, the minimum retracting force of the gel is measured. According to Figure 5a,d, the growth of pH led to an increase in firmness (viscosity) of the gel and, accordingly, an increase in minimum force, which is necessary for the separation of the gel from contact surface that also characterizes the adhesiveness of the gel.

**Figure 6:**
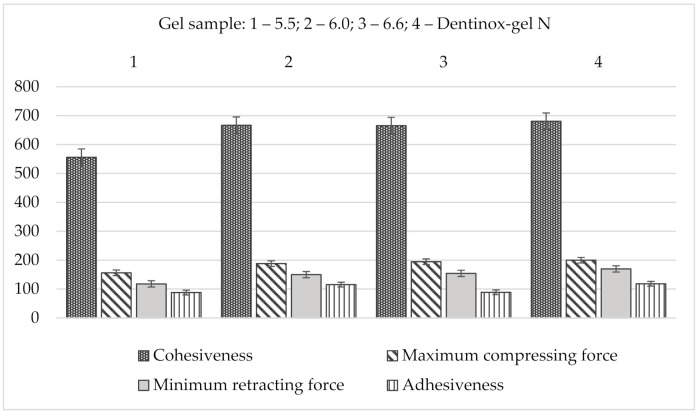
Comparison back extrusion test parameters of gel samples with “Dentinox-gel N” (*n* = 3).

The results of back extrusion analysis of gel’s samples in comparison with “Dentinox-gel N” are presented in Figure 6.

Summarizing the results of the back extrusion measurement, all samples of the test gel were characterized by the close resemblance to the reference drug. According to all studied indicators, especially in terms of adhesiveness, the sample with a pH 6.0 had the closest values to the “Dentinox-gel N” preparation.

**Figure 7:**
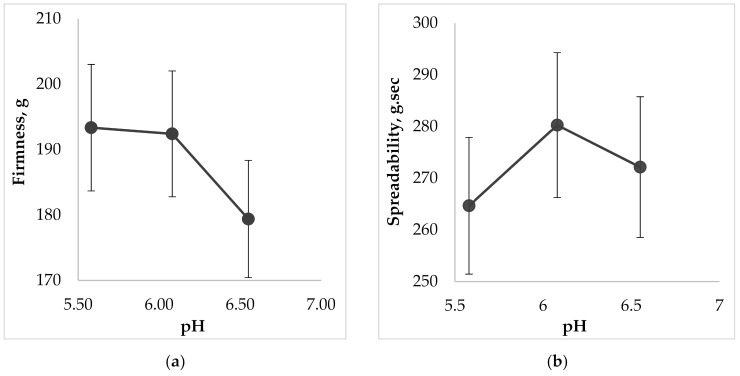

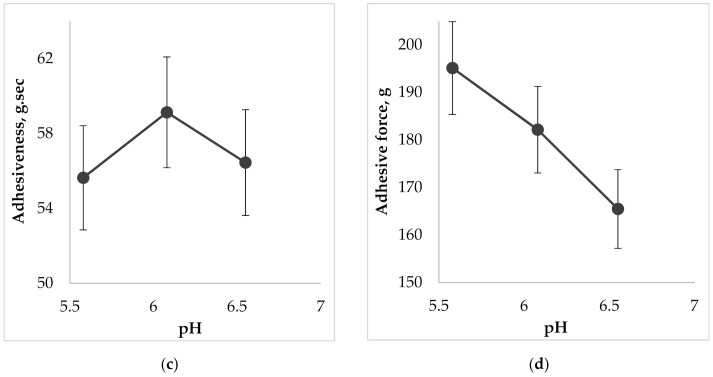
The effect of pH on the gel characteristics during the determination of spreadability: (**a**) on firmness; (**b**) on spreadability; (**c**) on adhesiveness; (**d**) on adhesive force.

Another important property for topical dental gels regarding their application on oral mucosa is spreadability. The measurements of this parameter were also carried out using Texture Analyzer, equipped with special conically shaped probes. When a probe penetrates into the sample, the device imitates a person’s finger, which touches the surface. The results of the spreadability test, including the analysis of this parameters dependence on the gel’s pH are shown in Figure 7.

According to the results of Figure 7 the more liquid the consistency of the sample, the more deformable it is, so measured firmness, spreadability, and adhesiveness varied within our limited range. The less viscous gel would be more liquid and wouldn’t retain its structure due to destruction of cohesive bonds in the system, and vice versa, more force is needed to spread a more viscous sample and to obtain a homogeneous layer of the medicine on the surface. The results of Figure 7d show that increasing of pH causes the decreasing of adhesive force, i.e., the interaction between molecules inside the gel is lower (low adhesiveness) and the repulsive force from the probe’s surface is higher. The lowest adhesive force was measured for the sample containing the pH value 6.6 (*p* < 0.05).

**Figure 8:**
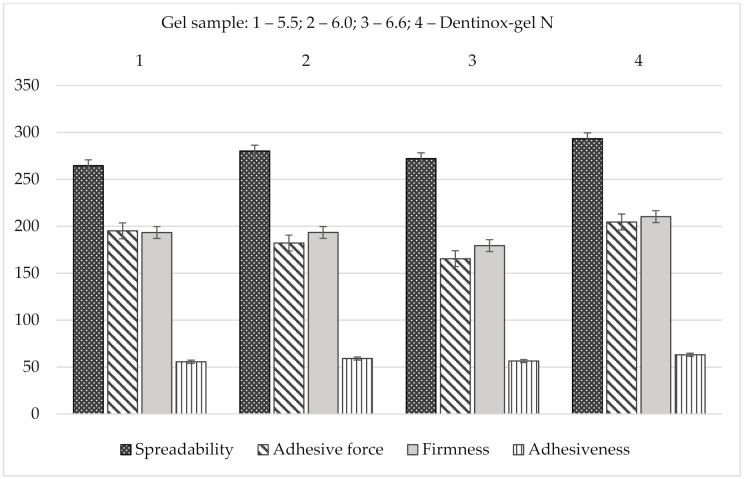
Comparative spreadability analysis of gel samples with “Dentinox-gel N” (*n* = 3).

The results of spreadability analysis of the gel’s samples in comparison with “Dentinox-gel N” are shown in Figure 8.

As shown in Figure 8, “Dentinox-gel N” had highest firmness, spreadability, adhesiveness, and adhesive force values (*p* < 0.05) comparing with the tested carbomer gels. According to indicators of spreadability and adhesiveness, the gel sample with pH 6.0 was also the most similar to the reference preparation.

**Figure 9:**
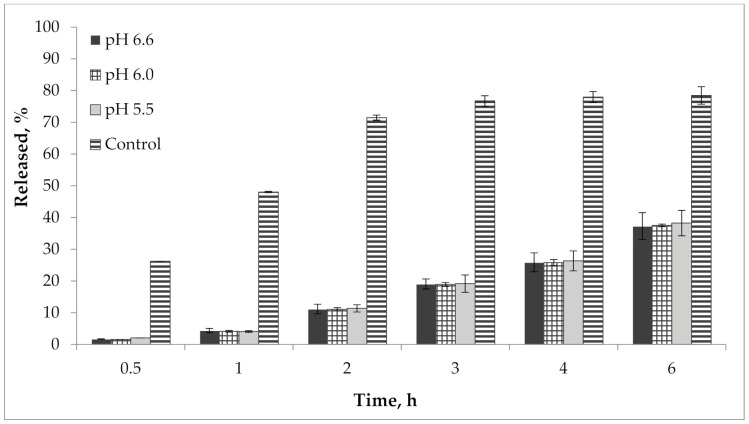
Kinetics of choline salicylate release from gel samples in comparison with control solution (*n* = 3).

**Figure 10:**
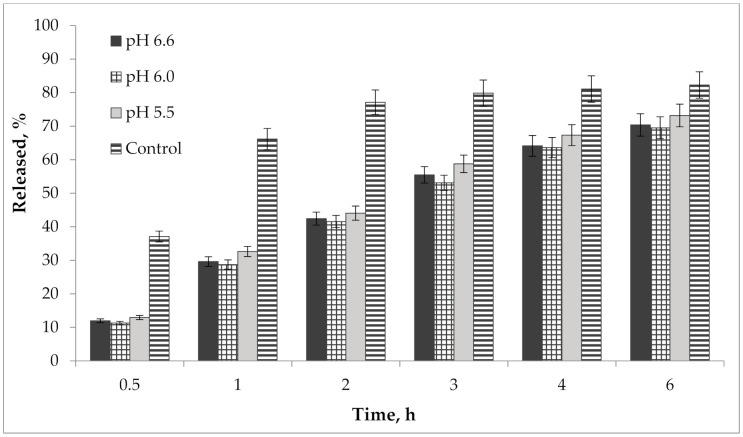
Kinetics of lidocaine hydrochloride release from gel samples in comparison with control solution (*n* = 3).

In addition, we performed a study of the pH effect on the release of active compounds from the gel. Bioavailability of the developed semisolid drug was established in vitro according to the results of the release dynamics study of choline salicylate (ChSal) and lidocaine hydrochloride (LidHyd) from the gel samples with different pH using the method of dialysis through a semi-permeable membrane [42,43]. HPLC technique was used to quantify choline salicylate and lidocaine hydrochloride. Aqueous solutions of the studied APIs in concentrations corresponding to their content in the drug were used as control samples. Dialysate samples were taken at 0.5, 1, 2, 3, 4 and 6 h after the start of the experiment. The obtained data is shown in Table 2 and in Figure 9 and Figure 10.

According to the data presented in Table 2 and in Figure 9 and Figure 10, an equal increase in the quantity of APIs in the dialysate was observed over the duration of the 6 h study, indicating a prolonged release of choline salicylate and lidocaine hydrochloride from the gel samples. Choline salicylate and lidocaine hydrochloride from the control solution are completely released in 3 h, i.e., prolongation is not due to membrane-sorbtion effect. However, according to the results, there is no statistically significant difference between the samples, i.e., the change in pH has practically no effect on the release of APIs from the developed carbomer-based gel.

## 3. Materials and Methods

### 3.1. Materials

A combination of APIs, namely the tincture “Phytodent” (PJSC “CPP Chervona Zirka”, Kharkiv, Ukraine), choline salicylate 80% (BASF Pharma, Evionnaz, Switzerland) and lidocaine hydrochloride (Societa Italiana Medicinali Scandicci, Firenze, Italy) were used in the development of the dental gel. The tincture “Phytodent” is a medicine containing a complex mix of plant-based active components (Acorus (*Acorus calamus* L.) rhizomes, Japanese Sophora (*Styphnolobium japonicum* L.) and Rose (*Rosa* L.) hips, Calendula (*Calendula officinalis* L.) and Chamomile (*Matricaria recutita* L.) flowers, Celandine (*Chelidonium majus* L.) grass, Nettle (*Urtica dioica* L.) leaves) and has anti-inflammatory, antiseptic, fungicidal, reparative and hemostatic effects. Carbomer Polacril^®^ 40P as a gel former was supplied by Amedeo Brasca & C. Srl (Origgio, Italy). Sodium hydroxide, from which a 10% solution was prepared and used as a neutralizing agent, was purchased from State Plant for Chemical Reagents (Kharkiv, Ukraine). Propyl parahydroxybenzoate (Nipasol M) and Methyl parahydroxybenzoate (Nipagin M) as antimicrobial preservatives were supplied by Clariant (Frankfurt, Germany). Vinyl methyl ether and maleic anhydride copolymer (OraRez^®^ W-100L16) as mucosal adhesive was received from Boai NKY Pharmaceuticals Ltd. (Henan, China) as a gift sample. The dental gel samples were prepared using purified water as a solvent. All other chemicals used in experiments were of analytical grade.

The composition of the analyzed dental gel is given in Table 3.

### 3.2. Gel Preparation

To obtain the gel, Carbomer Polacril^®^ 40P was dispersed in purified water at room temperature while constantly stirring for 1–2 h. Solution of APIs was prepared separately: methyl parahydroxybenzoate, propyl parahydroxybenzoate, lidocaine hydrochloride, and choline salicylate were dissolved in “Phytodent” tincture. As a result, a homogeneous transparent light-brown solution was obtained. After continued stirring, OraRez^®^ W-100L16 to carbomer dispersion was introduced. This mixture was neutralized by the 10% solution of sodium hydroxide to the required pH and then the solution consisting of APIs was added. Subsequently, the homogeneous gel of light brown color was obtained.

### 3.3. Methods

#### 3.3.1. Rheological Studies

Measurement of the rheological parameters were carried out using a rotational coaxial cylinder viscometer in accordance with the requirements of Ph.Eur. (current edition) chapter 2.2.10 [44].

Evaluation of the rheological parameters was carried out through two different means—by using a Rheolab QC viscometer with a coaxial cylinder (“Anton Paar”, Graz, Austria), as well as a viscometer MCR102 (“Anton Paar”, Graz, Austria) equipped with a geometry “plate-plate”.

In the first setup, we took an experimental sample weighing 17.0 g, which was placed in a gap between the inner and outer cylinders. The indicators of the viscometer were recorded at each speed of the inner cylinder after reaching stable indicators. Determination was performed by rotating the inner cylinder at different shear rates, from small to large and backwards [45,46,47,48]. The results were used to construct reograms showing the dependence of the tangential shear stress (τ_r_) and structural viscosity (η) on the velocity gradient (D_r_). The study was carried out at (25 ± 0.1) °C.

In the second setup, the sample of gel, which weighed about 1.0 g, was placed in the gap of 1 mm between two flat disks (plates). Rheological parameters were measured at (25 ± 0.1) °C and (37 ± 0.1) °C by rotating the upper disk at different shear rates, from small to large and backwards (similar to method 1). Temperature was controlled using a Peltier system. The results were used to construct a rheogram of the viscosity vs. shear rate in the range of 0.01 to 100 s^−1^

#### 3.3.2. Textural Analysis

A texture analyzer TA.XT Plus (Stable Micro Systems Ltd., Surrey, United Kingdom) was used to determine the texture properties of the gel samples.

##### Back Extrusion Test

**Figure 11:**
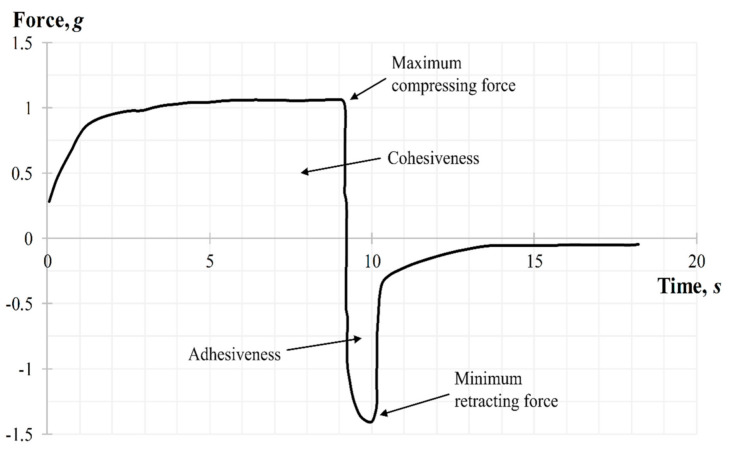
Back extrusion graph during the texture analysis.

The back extrusion test was performed using the Back Extrusion Rig A/BE. Approximately 50 mL of gel was placed in a standard sample container with a capacity of 100 mL, in a way to avoid air entering and providing a smooth upper surface. The gel was compressed by a disk with 40 mm of diameter, which was preliminary installed above the surface of the sample, providing an extrusion of the product upward between the walls and edges of the disk. The parameters of the study, including speed rate (2 mm/s) and distance (depth of insertion) (10 mm), were chosen. Three replicate analyses were carried out at a room temperature for each sample, providing the same conditions for each measurement. The gel parameters, such as the firmness (maximum compressing force), cohesiveness, adhesiveness, and minimum retracting force were determined from the force–time graph. A typical graph for measuring the back extrusion force for carbomer-based hydrogels is shown in Figure 11. When the probe with the disk moves down the positive part of the back extrusion graph the maximum compressing force is created, which is required for the gel deformation, showing the firmness of the gel form, while the area of the graph above zero demonstrates the cohesiveness of the gel. The higher the value, the denser is consistency of the sample. Once the disk is returned to its starting position, its upward movement creates a negative part of the graph: the area below zero shows adhesiveness and sample resistance during separation from the disk (minimum retracting force of gel). The higher the value, the more energy is required to break the contact of the sample with the disk surface, and, accordingly, the better is the adhesiveness of the gel.

##### Spreadability Test

**Figure 12:**
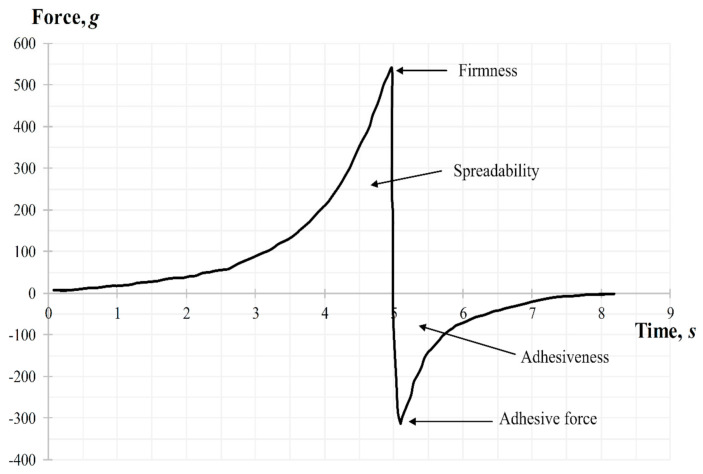
Spreadability graph during the texture analysis.

The ‘Gel spreading’ test was performed using the TTC Spreadability Rig HDP/SR. The gel sample was placed, avoiding air entering and providing a smooth upper surface, in a cone-shaped receiver. The probe, which is also a cone-shaped, was preliminarily installed above the surface of the gel. Parameters of the study were chosen: movement speed—2 mm/s, distance (depth of probe insertion into the gel)—10 mm. Three replicate analyses were performed at room temperature for each sample, providing the same conditions for each measurement. Parameters of gel such as firmness, spreadability, and adhesiveness were determined from the graph “force-time” (Figure 12). During the test (downward movement of the probe) the gel should flow outward between the surfaces of the cone-shaped receiver and the probe at an angle of 45°. The ease of this process indicates the gel has a high level of spreadability (area above zero). The peak of the positive part of the graph shows the gels ability to flow (firmness). The higher the value—the more liquid consistency the sample has, which also negatively affects the spreadability of gel. Removal of the cone probe from the sample (upward movement) gives the information about the adhesiveness of the gel. The ease of sample separation from the probe surface is shown by the maximum peak on the negative part of the graph (the value of the adhesive force).

#### 3.3.3. Release Test in Vitro

A USP 4 apparatus (Sotax CE7 smart with Sotax CP 7-35 pomp, Sotax AG, Switzerland) was used for in vitro release testing [42]. Flow through cells (22.6 mm diameter) with adapter for semisolids were used in a closed system. The system was temperature controlled at 32 ± 0.5 °C. Approximately 1.2 g of tested formulations were added to the adapter and fitted with regenerated cellulose membrane Cuprophan (Medicell International Ltd., London, Great Britain). 50 mL of distilled water was circulated at a flow rate 16 mL/min (120 pulse/min). One ml samples were withdrawn and replaced with fresh media at suitable time intervals. The studies were repeated thrice for each gel sample. The samples were diluted and measured by HPLC.

##### HPLC Analysis

Analysis was performed using a Shimadzu Nexera X2 LC-30AD HPLC system (Shimadzu, Tokyo, Japan) composed of a quaternary pump, an on-line degasser, a column temperature controller, the SIL-30AC autosampler (Shimadzu, Tokyo, Japan); the CTO-20AC thermostat (Shimadzu, Tokyo, Japan) as well as the SPD-M20A diode array detector (DAD) and a chromatographic column ACE 5 C18 with particle size of 5 µm (250 × 4.6 mm) with a pre-column. Acetonitrile (HPLC grade, Sigma-Aldrich GmbH, Switzerland), HPLC grade water that was obtained from a water purifying system (Millipore, Bedford, MA, USA), sodium phosphate dibasic and phosphoric acid (Sigma-Aldrich GmbH, Switzerland) were used in the analysis work.

The chromatographic conditions: a mobile phase A—acetonitrile, a mobile phase B—3.0 g/L sodium phosphate dibasic solution adjusted to pH 3.0. The chromatography was carried out by the following gradient program: 0–3 min 25% A, 75% B; 3–13 min 25→50% A, 75→50% B; 13–20 min 50% A, 50% B; 20–21 min 50→25% A, 50→75% B; 21–25 min 25% A, 75% B. The flow rate—1 mL/min, the injection volume—10 µL, detection at the wavelength of 260 nm, the column temperature −30 °C.

#### 3.3.4. Statistical Analysis

The results are presented as mean ± standard deviation. Statistical analysis was performed using Student’s *t*-test. A value of *p* < 0.05 was taken as the level of significance.

## 4. Conclusions

The effectiveness of topical dental gels is closely related to its rheological and textural properties, which directly influence the outcome of periodontal disease therapy and the consumption properties of the drug. The samples of Carbomer Polacril^®^ 40P-based dental gel, neutralized to different pH values, exhibited viscoelasticity, plastic behavior (flow index (*n*) ranged from 0.12 to 0.14), almost complete absence of thixotropy, and resistance to storage (25 °C) and application (37 °C) temperatures; their structural viscosity increases with growing pH values. Texture analysis revealed the consistency properties (viscosity (firmness), cohesiveness), spreadability, and adhesiveness. The interrelation between these parameters and their dependence on the pH change of the studied samples were established. The texture test of the dental gels proved a strong correlation with rheological parameters.

Study on the rheological, textural and release properties of the samples revealed that the behavior of the developed gel in the pH range 5.5–6.6 hasn’t changed significantly.

The relatively narrow range of working pH values did not significantly affect the viscosity of the preparation, or consequently the release of APIs from the developed Carbomer Polacril^®^ 40P-based dental gel.

## Figures and Tables

**Table 1:** Thermorheological characteristics of gel samples (*n* = 3).

Gel Sample	Consistency Index (*K*)		Flow Behavior Index (*n*)	
	25 °C	37 °C	25 °C	37 °C
pH 5.5	65.31 ± 2.36	58.26 ± 2.01	0.134 ± 0.005	0.132 ± 0.005
pH 6.0	80.83 ± 1.58	78.83 ± 1.05	0.127 ± 0.002	0.124 ± 0.002
pH 6.6	97.57 ± 2.04	95.27 ± 1.74	0.126 ± 0.004	0.123 ± 0.002
Dentinox-N	74.04 ± 1.75	70.04 ± 1.75	0.129 ± 0.004	0.125 ± 0.003

**Table 2:** Kinetics of APIs release depending on time in comparison with the control solution (*n* = 3).

Gel Sample	API	Released amount of APIs, mg/cm^3^					
		Samples Selection Time, h					
		0.5	1	2	3	4	6
pH 5.5	ChSal	1.350 ± 0.165	2.680 ± 0.730	7.491 ± 1.762	12.637 ± 2.025	17.350 ± 2.581	25.201 ± 2.780
	LidHyd	0.233 ± 0.011	0.587 ± 0.027	0.793 ± 0.038	1.058 ± 0.047	1.212 ± 0.056	1.317 ± 0.061
pH 6.0	ChSal	1.055 ± 0.172	2.680 ± 0.331	7.215 ± 0.398	12.338 ± 0.606	16.884 ± 0.263	24.440 ± 0.360
	LidHyd	0.203 ± 0.009	0.517 ± 0.025	0.748 ± 0.033	0.956 ± 0.041	1.145 ± 0.054	1.251 ± 0.059
pH 6.6	ChSal	1.105 ± 0.035	2.822 ± 0.429	7.194 ± 0.972	12.261 ± 1.019	16.663 ± 1.915	24.015 ± 2.744
	LidHyd	0.215 ± 0.010	0.533 ± 0.026	0.764 ± 0.035	0.998 ± 0.044	1.154 ± 0.056	1.267 ± 0.060
Control	ChSal	17.262 ± 0.141	31.630 ± 0.510	47.076 ± 1.044	50.545 ± 1.123	51.342 ± 1.811	51.671 ± 2.012
	LidHyd	0.668 ± 0.029	1.191 ± 0.057	1.388 ± 0.066	1.437 ± 0.070	1.459 ± 0.071	1.481 ± 0.071

Note. Number of tests *n* = 3 for each formulation, *p* < 0.05.

**Table 3:** Composition of the analyzed dental gel.

Ingredients	Amount, %
Tincture “Phytodent”	15.0
Choline salicylate 80%	8.0
Lidocaine hydrochloride	1.5
Carbomer homopolymer (Polacril^®^ 40P)	1.5
Sodium hydroxide (10% solution)	to the required pH
Vinyl methyl ether and maleic anhydride copolymer	
(OraRez^®^ W-100L16)	1.5
Methyl parahydroxybenzoate	0.15
Propyl parahydroxybenzoate	0.05
Purified water	Up to 100.0
